# A robust response to combination immune checkpoint inhibitor therapy in HPV-related small cell cancer: a case report

**DOI:** 10.1186/s40425-018-0348-4

**Published:** 2018-05-09

**Authors:** Won Jin Ho, Lisa Rooper, Sarah Sagorsky, Hyunseok Kang

**Affiliations:** 1 Department of Oncology, Johns Hopkins University School of Medicine, 1650 Orleans St, CRB1 Rm#186, Baltimore, MD 21287 USA; 2 Department of Pathology, Johns Hopkins University School of Medicine, 410 N Broadway, Rm#2249, Baltimore, MD 21287 USA; 3 Department of Oncology, Johns Hopkins University School of Medicine, 1650 Orleans St, CRB1 G86, Baltimore, MD 21287 USA; 4 Department of Oncology, Johns Hopkins University School of Medicine, 1550 Orleans St, CRB2 5m44, Baltimore, MD 21287 USA

**Keywords:** Immunotherapy, HPV, Small cell cancer, Oropharyngeal

## Abstract

**Background:**

Human papillomavirus-related small cell carcinoma of the head and neck is an extremely rare, aggressive subtype with poor outcomes. Therapeutic options are limited and are largely adopted from small cell lung cancer treatment paradigms.

**Case presentation:**

This report describes a 69-year old male who was diagnosed of HPV-related oropharyngeal cancer with mixed small cell and squamous cell pathology which was clinically aggressive and progressed through multimodal platinum-based therapies. Upon manifestation of worsening metastatic disease, the patient was initiated on a combination of ipilimumab and nivolumab. Within 2 months of starting immunotherapy, a robust partial response was observed. During the treatment course, the patient developed immune-related adverse effects including new diabetes mellitus, colitis, and hypothyroidism. The disease-specific survival was 26 months.

**Conclusion:**

Combination immunotherapy may be an attractive option for HPV-related small cell head and neck cancers resistant to other treatment modalities and thus warrants further evaluation.

**Electronic supplementary material:**

The online version of this article (10.1186/s40425-018-0348-4) contains supplementary material, which is available to authorized users.

## Background

Human papillomavirus (HPV)-related small cell cancer of the head and neck (H&N) is extremely rare and has previously been reported as a distinct morphological variant [1, 2]. While HPV-related H&N squamous cell cancers have been associated with more favorable treatment response and survival rates when compared with HPV-negative cancers [3, 4], HPV-related H&N cancers with small cell morphology tend to progress aggressively with many cases demonstrating less than 15 months of disease-specific survival [1, 2, 5].

Given the rarity of this subtype, there are no preceding clinical trials providing for evidence-based guidelines for therapy. Thus, the treatment paradigm for small cell H&N cancers is largely derived from experience with small cell lung cancers (SCLC). Despite the adoption of platinum-based regimen, in combination with radiation therapy when appropriate, treatment outcomes for small cell H&N cancers overall remain poor without clear options for subsequent lines of therapy [6, 7]. Notably, immunotherapy in recent years have demonstrated promise in treating recurrent or metastatic H&N squamous cell cancers [8] as well as SCLC [9], the treatment options for which have historically been very limited.

Here, we present a case of metastatic H&N small cell cancer associated with HPV responding to combination immunotherapy after failing platinum-based chemotherapy with radiation.

## Case presentation

**Fig. 1:**
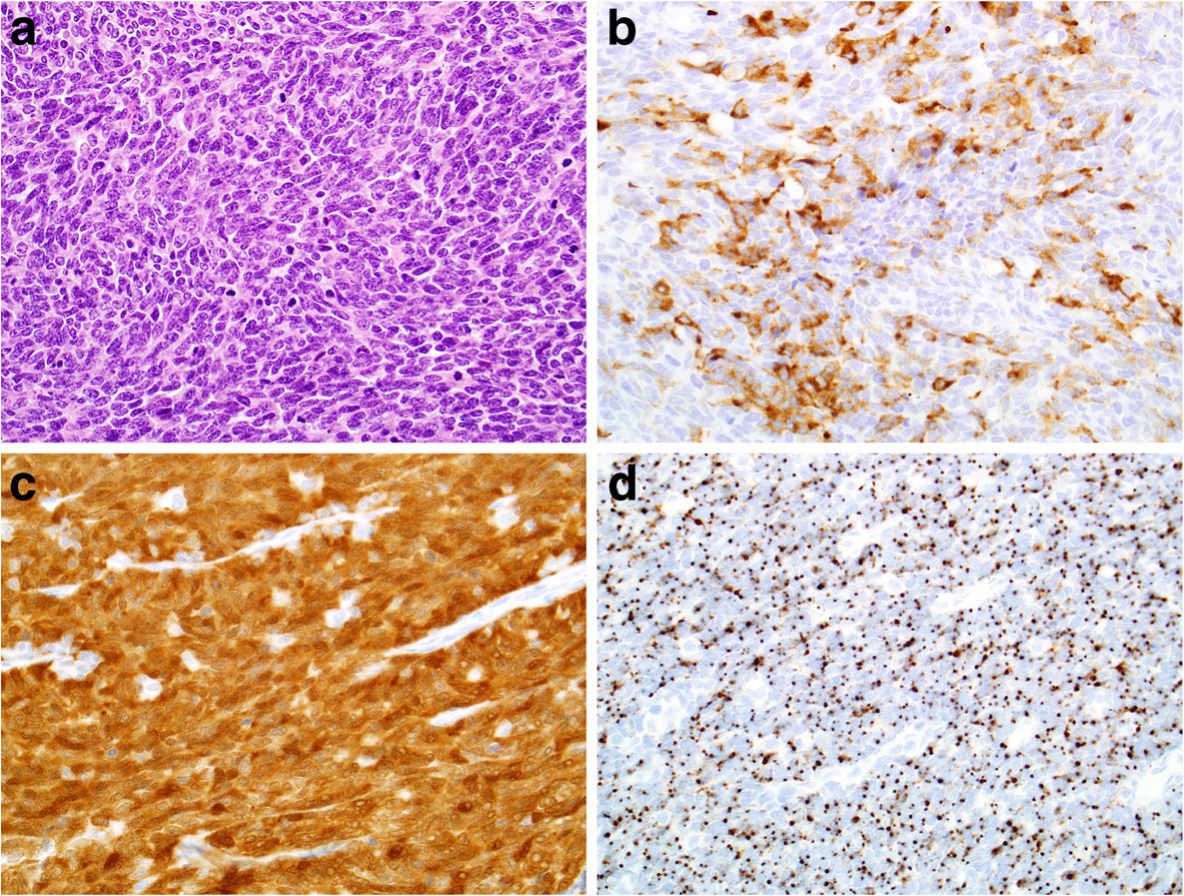
Hematoxylin and eosin stained sections demonstrated a population of tumor cells with minimal cytoplasm, a high nuclear-cytoplasmic ratio, speckled chromatin, nuclear molding, and prominent mitotic and apoptotic figures, consistent with small cell carcinoma (**a**, 400×). These cells demonstrated patchy positivity for the neuroendocrine marker synaptophysin (**b**, 400×) and diffuse, strong nuclear and cytoplasmic positivity for p16 (**c**, 400×) by immunohistochemistry as well as punctate nuclear reactivity for high-risk HPV by RNA in-situ hybridization (**d**, 400×), supporting the diagnosis of HPV-related small cell carcinoma

A 69-year old male patient who has a 30-pack-year smoking history presented with a left-sided neck mass which was increasing in size for several weeks. Fine needle aspiration (FNA) revealed a high grade basaloid carcinoma with myoepithelial and focal neuroendocrine features. He underwent direct laryngoscopy and left selective neck dissection levels II-IV which revealed pT1pN2bM0 glossopharyngeal sulcus carcinoma with mixed squamous cell and small cell pathology (focally positive for CD56, synaptophysin, and chromogranin) positive for high-risk HPV (Fig. 1). Genomic profiling of the tumor was performed via FoundationOne™. The following genomic alterations were detected: *KRAS* G12C, *TSC2* T1181 M, *EP300* R1055*, *LRP1B* loss exons 8–9 and loss exons 12–20, *CREBBP* S302 N, *MED12* A1753V, *PALB2* D255N, *PIK3R2* E342D, *STAT4* T446I, microsatellite stable disease, and tumor mutation burden of 5.59 Mut/Mb; no clearly actionable mutations were identified.

**Fig. 2:**
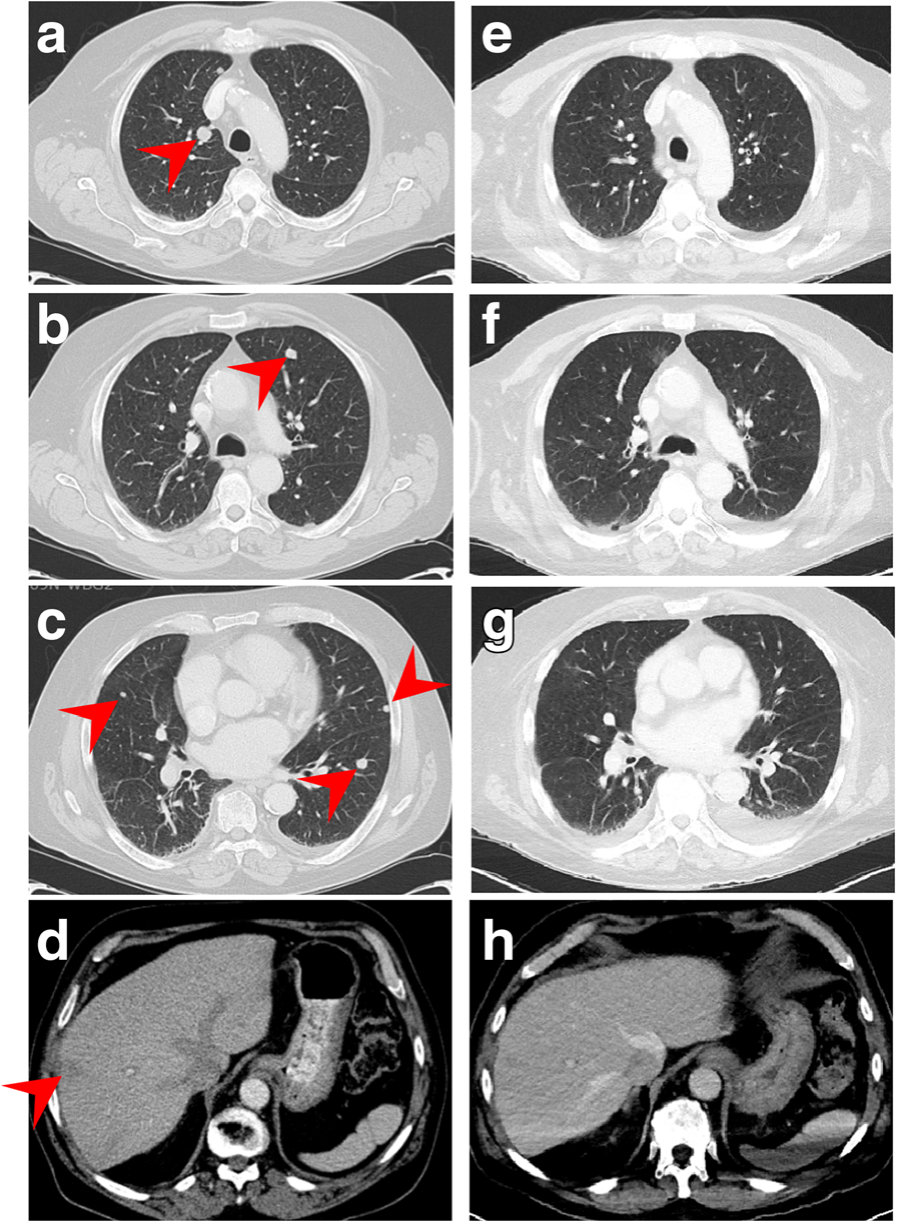
CT scans of the chest and abdomen show numerous bilateral pulmonary nodules measuring up to 43 mm (**a**-**c**) and liver lesions with a prominent 20 mm lesion (**d**) prior to immunotherapy initiation. All lesions are markedly improved (**e**-**h**) by four months after treatment with ipilimumab and nivolumab has started

Based on multidisciplinary tumor board review, decision was made to pursue chemoradiation over transoral robotic surgery/radical tonsillectomy. Adopting the treatment paradigm for small cell lung carcinomas, the patient underwent four cycles of cisplatin and etoposide with concurrent intensity-modulated radiation therapy for a total of 70Gy. During follow up, only minimally progressive changes were observed on the post-treatment scan and a three-month follow up scan. Subsequently, however, marked disease progression was noted in another 3 months with several new pulmonary nodules and a new hepatic metastasis confirmed by fine-needle aspiration. Despite four more cycles of palliative cisplatin and etoposide, post-treatment imaging demonstrated worsening pulmonary and hepatic disease (Fig. 2a-d).

**Fig. 3:**
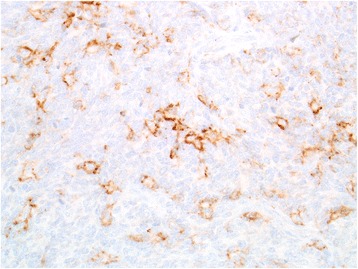
20% of tumor cells demonstrated membranous staining for PD-L1, consistent with low expression (400×)

Extrapolating the data from CheckMate 032 study involving patients with recurrent SCLC after failing platinum-based first-line therapy [9], combination immunotherapy regimen with ipilimumab 1 mg/kg every 6 weeks and nivolumab 240 mg every 2 weeks was initiated. To note, upon immunohistochemical analysis, the tumor demonstrated 20% positivity for PD-L1 expression via clone 22C3 antibody (Fig. 3). Further characterization of the tumor-infiltrating lymphocytes by immunohistochemistry revealed a robust presence of CD4+ cells, CD8+ cells, and Foxp3+ cells (Table 1). After one dose of ipilimumab and three doses of nivolumab, significant interval improvement in disease was noted on CT scans with decreased size of most metastatic lesions. After an additional dose of ipilimumab and nivolumab, the patient developed grade 3 immune-related colitis, hypothyroidism, and diabetes mellitus type I and was intermittently hospitalized. He was treated with high-dose steroid taper, which consisted of intravenous methylprednisolone 2 mg/kg IV for 1 week, followed by decreasing dose of oral prednisone every 4 days starting from 1 mg/kg for the next 4 weeks. He was also treated with levothyroxine, and insulin, and further immunotherapy was held thereafter. A repeat scan at 4 months from treatment initiation demonstrated complete resolution of a 20 mm hepatic lesion, complete resolution of pulmonary lesions that measured up to 23 mm, and 67% decrease in a pulmonary target lesion from 43 mm to 14 mm, all of which were consistent with continued partial response (2E-H). Unfortunately, he developed progression of disease in liver and lungs at 6-month restaging scans; progression-free survival was 6 months. He was re-challenged with single agent nivolumab with prophylactic vedolizumab, and tolerated four doses of the treatment well without worsening of immune-related colitis. However, he developed progression of disease with growing nodules in cervical LN, mediastinum, lungs, and liver as well as a singular brain lesion on the first restaging scan. The brain lesion was resected for palliation and diagnosis, confirming metastatic spread with positive PD-L1 expression and tumor-infiltrating lymphocytes (Table 1). The patient’s performance status rapidly declined in the following 2 months, was enrolled in hospice, and he passed away soon thereafter. Disease-specific survival at the time of death was at 26 months.

**Table 1:** Immunohistochemical analysis of tumor specimens

	PD-L1	CD4/HPF	CD8/HPF	FOXP3/HPF	CD8/FOXP3	CD8/CD4
Neck primary (pre-treatment)	20%	75	111	31	3.58	1.48
Metastatic brain (post-treatment)	10%	53	69	9	7.67	1.3

*HPF* high-power field; immunohistochemistry methods described in Additional file 1

## Discussion

### Literature review of HPV-related small cell cancers of H&N: A rare and aggressive disease

Small cell cancers of extrapulmonary primary have been reported to represent as much as 5% of all small cell carcinoma cases [10]. The existing treatment paradigms for SCLC have been accepted by many institutions as the first line approach for extrapulmonary small cell cancers [1, 2, 5–7, 10]. In fact, a single institutional review of 120 patients with extrapulmonary small cell cancer including H&N small cell cancers demonstrated sustained durable responses with multimodal therapy; 5-year survival of 25.4% for limited stage patients, a rate superior to most series of patients with limited stage SCLC [10]. However, as our patient also eventually progressed after platinum-based treatments with distant metastasis, the overall prognosis remains dismal even for HPV-positive cancers. Among five HPV-related oropharyngeal small cell cancer cases previously reviewed at Johns Hopkins Hospital by Bishop and Westra, three patients had disease-specific survival of only 6 to 15 months despite having been treated with chemoradiation (except for one patient who received chemotherapy only). The remaining two patients who underwent chemoradiation, one with surgical resection and one without, had reported follow up duration of 20 months and 5 months, respectively [1]. Kraft et al. also described their institutional experience, in which among four cases of HPV-related oropharyngeal small cell cancer with follow up information, three cases had total follow-up duration of 9 to 14 months, all ending with progression of disease in the setting of multimodal treatment regimens [2]. In another case series, two patients who were treated with carboplatin-etoposide and radiotherapy, as well as surgery in one patient, died due to progression with 15 and 29 months of disease-specific survival, respectively [5].

### Rationale behind the immunotherapy for HPV-related small cell cancer of the H&N

Recently, immunotherapy has emerged as a new attractive treatment modality in oncology. In small cell cancers of the lung, CheckMate 032 showed that nivolumab, a PD-1 inhibitor, with or without ipilimumab, a CTLA-4 inhibitor, provides durable responses for some patients who have failed one or more platinum-containing therapy [9]. In the field of H&N cancers, positive results from a nonrandomized trial exploring the use of pembrolizumab, also a PD-1 antibody, in patients with advanced solid tumors led to the first approval of a checkpoint inhibitor in the treatment of H&N squamous cell cancers [11]. Subsequently, CheckMate 141 demonstrated that nivolumab was preferable to standard single-agent options (methotrexate, docetaxel, or cetuximab) in platinum-refractory H&N squamous cell cancers with higher rate of overall survival and lower rate of treatment-related adverse effects [8]. Now several clinical trials are underway for H&N cancers to explore combinatorial approaches to inhibit not only PD-1/PD-L1 signaling but also in conjunction with anti-CTLA-4 agents. These efforts have thus provided the grounds for trialing our patient with combination immunotherapy.

However, the exact mechanism by which the combination of nivolumab and ipilimumab have provided their therapeutic efficacy in our case of HPV-associated H&N small cell cancer is not clear. In principle, inhibiting immunoregulatory pathways induces antitumor immune responses within the tumor microenvironment, but it remains largely speculative whether the intrinsic microenvironmental characteristics associated with the histologic phenotype or HPV status have actually contributed to the therapeutic effect seen in our case. As for small cell cancers, Antonia et al. observed that PD-L1 expression in SCLC was overall less prevalent than in squamous cell lung cancers but that PD-L1 expression status did not necessarily predict tumor responses to PD-1 inhibition [9]. In fact, in our case, there was 20% positivity for PD-L1 expression. Thus, whether the mixed morphology involving squamous phenotype had any bearing on the overall response of the tumor to the immunotherapy is unknown. However, with regards to HPV status in the H&N cancers, HPV-related H&N squamous cell cancers were associated with better responses to nivolumab than HPV-negative tumors [8]. Furthermore, an immune-landscape analysis demonstrated that HPV-positive H&N tumors possess significantly higher CTLA-4 expression and presence of infiltrating regulatory T cells than HPV-negative H&N tumors [12]. Based on our immunohistochemical analysis, both the pre-treatment tissue from the neck and the post-treatment tissue from the brain demonstrated positive PD-L1 expression and substantial presence of CD8+ cells. Prior studies have shown that CD8/Foxp3 and CD8/CD4 ratios are significantly greater in HPV+ than HPV- HNSCC, suggesting a relatively heightened immunologic microenvironment in HPV+ disease [13, 14]. While studies have demonstrated that higher presence of CD8+ cells correlate with a more favorable prognosis in both HNSCC and SCLC, there is only limited evidence that pre-treatment presence of CD8+ T-cells predicts response to checkpoint inhibitor therapy [15, 16].

Taken together, we suspect that such a highly immune-mediated tumor environment, especially as related to HPV infection, may have provided the context in which inhibition of both CTLA-4 and PD-1/PD-L1 pathways led to significant antitumor effects.

### Insights from the genomic profile

Genomic analysis of SCLC revealed that inactivating mutations in *TP53* and *RB1* affect the vast majority of all tumors, suggesting the notion that the loss of function in these two genes is obligatory in the carcinogenesis of SCLC [17]. Among other genes recurrently mutated in SCLC, mutations in *CREBBP* and *EP300* histone acetyletransferases have also been identified [17]. Given that the fundamental oncogenic process of HPV infection features viral oncoproteins E6 and E7 interfering with the function of p53 and Rb proteins, respectively [18], it was not entirely unexpected that the genomic sequencing of our patient’s HPV-related tumor did not reveal mutations in *TP53* and *RB1*. Notably, the cancer was positive for mutations in *CREBBP* and *EP300*. These observed alterations together are consistent with and provide a potential molecular basis for the small cell phenotype seen in our case.

Tumor mutational burden and DNA repair deficiencies have been suggested as predictive biomarkers for immune checkpoint inhibitor therapy; tumors that have higher mutational load and/or poor DNA repair mechanisms presents more neoantigens, thereby becoming more susceptible to recognition by the immune system [19]. Furthermore, while HPV viral antigens may serve as neo-epitopes, a recent report has suggested that immune activation against nonviral tumor antigens rather than canonical viral antigens in fact plays a major role in anti-tumor activity [20]. Le et al. demonstrated that tumors with mismatch repair (MMR) deficiency regardless of their tissue of origin are sensitive to immune checkpoint inhibition [21]. In this study, neuroendocrine tumors were among the tumors that had higher rate (> 5%) of MMR deficiency. Also notable, in cataloguing the somatic mutational signatures of cancers, Alexandrov et al. previously observed that both SCLC and head and neck cancers possess relatively higher mutational burdens among 30 human cancer types compared [22]. Interestingly, genomic profiling of our patient’s cancer identified a mutation in *PALB2*, a gene intimately involved in DNA repair machinery, suggesting a potential genetic basis for immunotherapy response. However, the assay also detected low tumor mutational burden in the cancer, when estimated by targeted comprehensive genomic profiling [23]. Overall, it remains unclear what impact these genetic characteristics truly had on the observed response to immunotherapy.

### Potential significance of immune-related adverse events

Lastly, our patient developed multiple immune-related adverse events (IRAE). To date, it has not been determined whether a particular type of IRAE or the severity of IRAE portends significant treatment response, but correlations have been observed in other cancers. For example, in melanoma, multiple reports have associated cutaneous IRAE such as vitiligo with better immunotherapy-related treatment outcomes [24, 25], leading to the hypothesis that there may be lineage-specific epitopes shared by both the normal cells and tumor cells of the same tissue origin [26]. Additional studies to characterize and understand the potentially meaningful relationships between treatment outcomes and IRAE would be valuable.

Vedolizumab is a monoclonal antibody against integrin α4β7, which is primarily expressed on a subset of CD4+ T cells and mediates homing of these cells to GI tract [27]. It is approved for treatment of inflammatory bowel disease, and a case series have suggested that it is effective and well tolerated for patients with steroid refractory immune related colitis [28]. We chose to use vedolizumab prophylactically before re-challenge with nivolumab, as it is not expected to have profound systemic immune suppressive effects. Our patient did not develop immune related colitis, although he was taken off because of progression of disease.

## Conclusion

In this report, we have described a patient with HPV-related H&N small cell cancer, an aggressive and rare variant of HPV-related H&N cancers, who has demonstrated significant response to combination immunotherapy with ipilimumab and nivolumab after having progressed through the most adopted treatment approach involving radiation and combination chemotherapy with platinum and etoposide. Thus, immunotherapy regimens based on PD-L1 and/or CTLA-4 inhibition may be an effective approach to treating HPV-related H&N small cell cancers and should be considered for patients who are refractory to first lines of therapy. Taking into account the rarity of this cancer, additional experience and larger-scale studies focused on molecular characteristics of small cell cancers, HPV-related cancers, or the relevant tumor microenvironment features may be more productive in further establishing the utility of immunotherapeutic approaches and any potential predictive markers.

## Additional file

Additional file 1: Methods for immunohistochemistry analysis. (DOCX 12 kb)

## Abbreviations

CREBBP: CREB binding protein
CTLA-4: Cytotoxic T-lymphocyte associated protein 4
EP300: E1A binding protein p300
H&N: Head and neck
HPV: Human papillomavirus
IRAE: Immunotherapy related adverse effects
MMR: Mismatch repair
PD-1/PD-L1: Programmed cell death protein 1/programmed death-ligand 1
RB1 (Rb): Retinoblastoma 1
SCLC: Small cell lung cancer
TP53 (p53): tumor protein p53
