# Supplementary material for: A robust response to combination immune checkpoint inhibitor therapy in HPV-related small cell cancer: a case report
Source: J Immunother Cancer. 2018 May 9;6:33. doi: 10.1186/s40425-018-0348-4 (PMC5943998; doi:10.1186/s40425-018-0348-4)
Supplement: Supplementary file 1 — Methods for immunohistochemistry analysis. (DOCX 12 kb) [file 40425_2018_348_MOESM1_ESM.docx]

Additional File 1.

***Immunohistochemical analysis***: Immunostains for PD-L1 (clone 22C3; Dako, Carpinteria, CA), FOXP3 (clone 236A/E7; eBioscience, San Diego, CA), CD4 (clone SP35; Ventana Medical Systems, Tucson, AZ), and CD8 (clone C8144B; CellMarque/Sigma-Aldrich, St. Louis, MO) were performed. The percentage of tumor cells expressing membranous PD-L1 was evaluated manually across a whole-slide section of tumor. The number of inflammatory cells expressing FOXP3, CD4, and CD8 were manually quantitated and averaged across three representative high-power fields (HPF).
